# Prognostic and Diagnostic Significance of circRNA Expression in Esophageal Cancer: A Meta-analysis

**DOI:** 10.1155/2020/8437250

**Published:** 2020-12-01

**Authors:** Hong Lin, Jinpeng Yuan, Guoxi Liang, Yanxuan Wu, Liming Chen

**Affiliations:** ^1^Department of Oncology, The First Affiliated Hospital of Shantou University Medical College, Shantou, Guangdong, China; ^2^Shantou University Medical College, Shantou, Guangdong, China; ^3^Department of General Surgery, The First Affiliated Hospital of Shantou University Medical College, Shantou, Guangdong, China; ^4^Department of Radiation Oncology, Cancer Hospital of Shantou University Medical College, Shantou, Guangdong, China

## Abstract

**Background and Aims:**

Circular RNA (circRNA) demonstrates potential biological application in various solid tumors. We intended to evaluate the diagnostic, prognostic, and clinicopathological value of circRNA for esophageal cancer (EC).

**Methods:**

We screened relative studies from Pubmed, Embase, Web of Science, and Cochrane Library. The diagnostic role of circRNAs was testified by pooled sensitivity and specificity. Pooled odds ratio (OR) and pooled hazard ratio (HR) were computed to appraise the clinicopathological and prognostic value, respectively.

**Results:**

There were total 15 articles suitable with our included criteria, in which 7 for diagnosis, 8 for prognosis, and 9 for clinicopathological features. The pooled sensitivity and specificity were 0.77 and 0.80, respectively, while the AUC was 0.85. Patients with aberrant expression of circRNAs had a 2.92-fold increased risk of developing EC. The proportion of EC patients with normal circRNA expression only accounted for 29%. Upregulated expression of oncogenic circRNA was correlated with poor clinicopathological features, including lymph node metastasis, tumor size, and T classification, while downregulation of tumor-suppressor circRNA was contributed to worse TNM stage. As for prognosis, upregulated expression of circRNA carried out a diverse survival outcome, with a pooled HR of 2.76 for tumor promoter and that of 0.21 for tumor suppressor. High expression of oncogenic circRNA in both plasma and tumor tissue would lead to a shorter survival duration.

**Conclusion:**

circRNAs might be a promising biomarker for diagnosis, prognosis, and clinicopathological features of EC.

## 1. Introduction

Circular RNA (circRNA), consisting of a covalently closed circular structure without 5′ to 3′ polarity, is an endogenous noncoding RNA produced by unconventional splicing of pre-RNAs [1–3]. It was initially discovered by Sanger et al. [4] in 1976; then, it was proved to adsorb endogenous micro-RNAs (miRNAs) as miRNAs sponges in 2013 [5, 6]. Besides, circRNA plays roles in transcription, selective splicing regulation, cell cycle regulation, methylation modification, and information transport [7]. Other studies demonstrated that circRNA accelerated proliferation, differentiation, and apoptosis of tumor cell [8]. With the development of sequencing technology, circRNA was found to be of remarkable importance in various diseases, including cardiovascular disease, diabetes, Alzheimer's disease, and cancer [5, 7, 9, 10]. Due to its high conservatism and stability, circRNA might become a promising biomarker in cancer diagnosis and therapy.

Esophageal cancer (EC) is a common malignancy of the digestive tract with poor prognosis. In all malignancies, EC ranks among the top ten both in morbidity and mortality [11]. Adenocarcinoma and squamous cell carcinoma are two most frequent histologic types of EC. The former occurs mainly in developed countries, and the latter occurs mostly in Eastern Asia [12, 13]. Regrettably, EC is often diagnosed at middle-advanced stage, which results in the omitting of optimal therapeutic opportunity for patients. The primary reasons include the delayed emergence of initial symptoms, discomfort caused by endoscopy, and the nonspecific and insensitive tumor markers [14]. Recently, the role of epigenetics in esophageal cancer is gradually being discovered. The occurrence and progression of malignant tumors are usually first accompanied by changes in the microenvironment and signaling pathways, such as the regulation of the vascular network of EC by miR-126 and miR-377 [15]. Many studies discovered abnormal expression of circRNA in EC, which may provide crucial reference for diagnosis and treatment. We incorporated relevant studies for a meta-analysis, in order to summarize the correlation between circRNA expression and diagnosis, prognosis, and clinical characteristics of EC.

## 2. Materials and Methods

### 2.1. Search Strategy

Our research was carried out on the basis of the preferred reporting items for systematic reviews and meta-analyses (PRISMA) checklist (Supplementary file (available here)) [16]. We searched for studies from four online databases, including Pubmed, Embase, Web of Science, and Cochrane Library, by using the following terms: (1) (“esophageal carcinoma” or “esophageal cancer” or “esophageal tumor” or “esophageal neoplasm” and (2) (“circular RNA” or “circRNA”). The deadline for searching was June 17th, 2020. Two researchers (HL and JPY) evaluated the appropriate studies and extracted the imperative data independently. If there was any disagreement, a third researcher (LMC) together with HL and JPY would discuss and resolve it.

### 2.2. Study Selection

Studies that met the following eligibility were included into our meta-analysis: (1) patients were diagnosed as EC by positive histology, (2) the studies were performed to estimate the diagnostic or prognostic efficiency of circRNA for EC or to identify the relationship between the expression of circRNA and clinicopathologic features, and (3) cohort or case-control researches. The excluded criteria were listed as the following: (1) articles that were not published in English; (2) review, meta-analysis, letter, and animal studies; and (3) with incomplete information.

### 2.3. Data Extraction and Quality Assessment

Two researchers (HL and JPY) extracted the following information from each study independently: (1) first author, country, edition year, cancer and circRNA type, the number of samples, sample species, experimental method, and regulated signature of circRNA; (2) the follow-up duration of EC patients; (3) diagnostic specificity and sensitivity, the area under the receiver operating characteristic (ROC) curve (AUC), the value of true positive (TP), false negative (FN), true negative (TN), and false positive (FP); and (4) clinicopathological features including age, gender, smoking, drinking, TNM stage, T classification, lymph node metastasis, distant metastasis, tumor size, and differentiation. If the parameter of TP, TN, FP, and FN was not offered, we assessed it according to sample size, specificity, sensitivity, and AUC.

Two independent researchers (HL and JPY) performed quality assessment of the included studies by using the Newcastle-Ottawa Scale (NOS) [17]. A score no less than 6 was conferred with high quality for a study.

### 2.4. Statistical Analysis

Stata 15.0 was utilized to develop related statistical analysis. By combining the number of TP, TN, FP, and FN, the pooled specificity, sensitivity, diagnostic odds ratio (DOR), and negative and positive likelihood ratio (NLR and PLR) were calculated. Summary receiver operator characteristic (sROC) curve with AUC (the area under sROC) was plotted to evaluate the diagnostic value of circRNA. Pooled odds ratios (ORs) with 95% confidence intervals (CI) were utilized to assess the relationship between the expression of circRNA and clinicopathologic features. In addition, we estimated the prognostic value of circRNA for overall survival (OS) via using pooled hazard ratios (HRs). Subgroup analysis was performed to determine whether the aberrant expression of circRNA in plasma or tumor tissue had an impact on prognosis. *I*^2^ value and chi-squared test were used to evaluate heterogeneity. A <50% *I*^2^ value or a <0.10 *p* value was considered of no conspicuous heterogeneity, so a fixed-effect model was applicable. Otherwise, a random-effect model should be adopted [18]. Potential source of heterogeneity was investigated via sensitivity analyses. In addition, funnel plots and Begg and Egger's tests were established to estimate publication bias.

## 3. Results

### 3.1. Search Results

**Figure 1:**
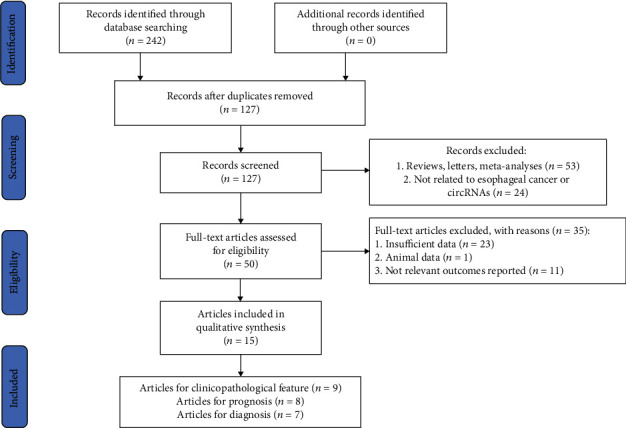
The flowchart of research selection.

The flowchart of study selection was plotted in Figure 1. A total of 242 articles were retrieved from online databases, in which 15 were suitable for being incorporated in the meta-analysis. There were seven [19–25] and eight [19–21, 23, 24, 26–28] articles on diagnostic accuracy and prognostic evaluation, respectively, while nine [20, 26–33] articles on clinicopathological parameter. Notably, Fan et al. found that both has_circ_0001946 and has_circ_0062459 were associated with the diagnosis of EC in their research. As a result, 8 datasets from 7 articles were adopted in analysis of diagnosis.

### 3.2. Study Characteristics and Quality Assessment

Tables 1 and 2 show us the basic characteristics of the included researches. A total of 16 kinds of circRNA, and 1032 participants were included. The individuals in each study ranged from 26 to 210. All studies were published from 2018 to 2020. The follow-up duration was from 20 to 90 months.  Table 1 shows the 8 datasets with sensitivity, specificity, and AUC. As Table 2 listed, 7 kinds of circRNA upregulated (tumor promoters) in EC, and 1 downregulated (tumor suppressors). The expression of circRNA was calibrated by quantitative real-time reverse transcription PCR (qRT-PCR). The sample specie for exploring diagnostic value of circRNA was plasma, while that for exploring clinicopathological features was tumor tissue. As for prognostic analysis, species included both plasma and tumor sample. What is more, the involved studies were of high quality (Table 3).

### 3.3. Diagnosis Analysis

**Figure 2:**
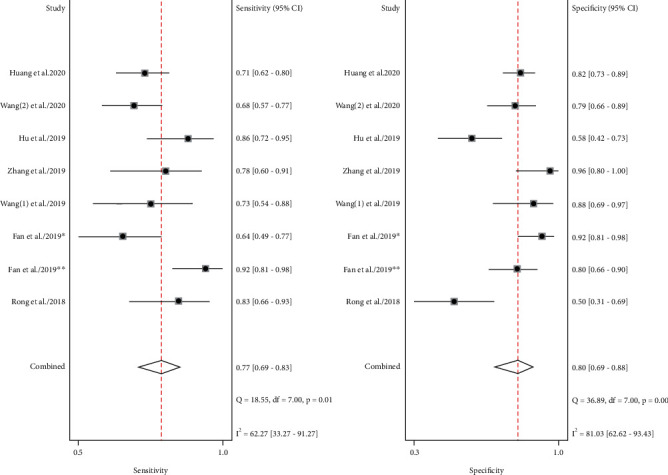
Forest plots of summary sensitivity and specificity to illustrate the diagnostic value of circRNAs for EC. circRNAs, circular RNAs; EC, esophageal cancer.

**Figure 3:**
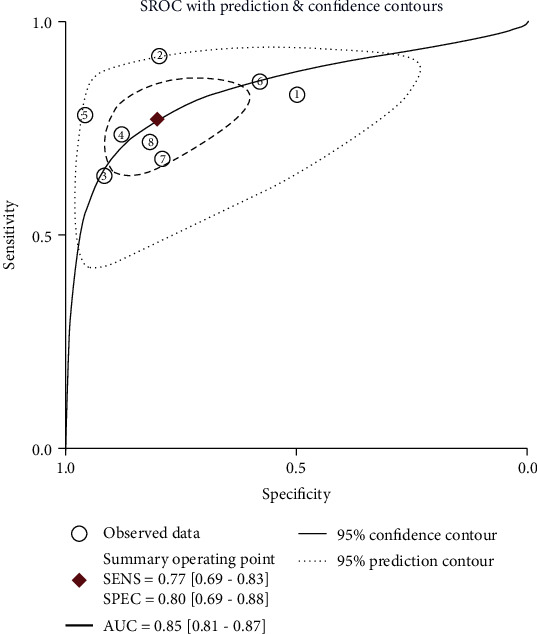
The summary ROC curve (sROC). ROC, receiver operator characteristic.

**Figure 4:**
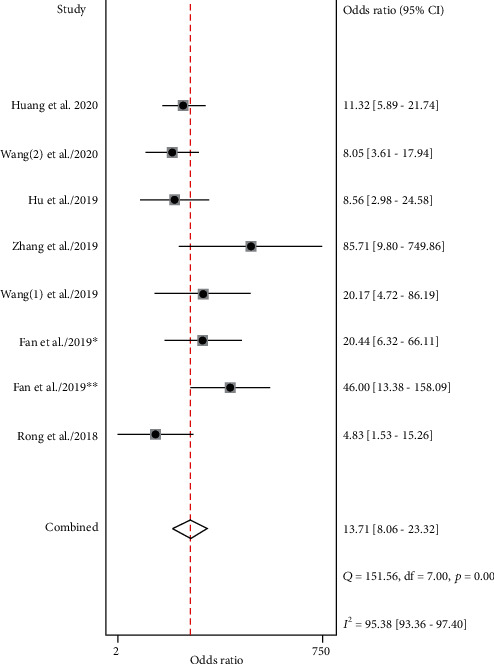
Forest plots of DOR of circRNAs for EC. DOR, diagnostic odds ratio; circRNAs, circular RNAs; EC, esophageal cancer.

**Figure 5:**
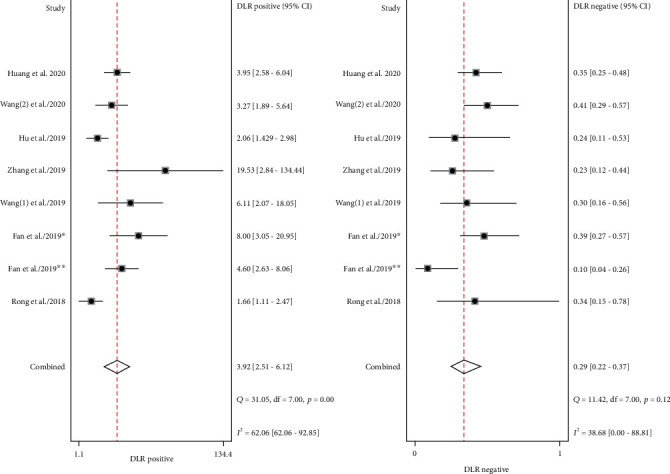
Forest plots of pooled PLR and NLP of circRNAs for EC. PLR, positive likelihood ratio; NLR, negative likelihood ratio; circRNAs, circular RNAs; EC, esophageal cancer.

There were 8 datasets from 7 articles finally incorporated into this meta-analysis. The forest plot demonstrated the pooled sensitivity and specificity of circRNA (Figure 2). Because of observable heterogeneity (*I*^2^ = 62.27% and *I*^2^ = 81.03%), a random-effect model was utilized. The calculated results revealed a pooled specificity of 0.80 (95% CI: 0.69–0.88) and a pooled sensitivity of 0.77 (95% CI: 0.69–0.83). The pooled AUC was 0.85 (95% CI: 0.82-0.88) (Figure 3). The conclusive DOR was 13.71 (95% CI 8.06-23.32) (Figure 4). Moreover, the pooled PLR was 3.92 (95% CI 2.51-6.12), and pooled DLR was 0.29 (95% CI 0.22-0.37) (Figure 5). Aforementioned outcomes demonstrated that circRNA could be a precise biomarker for EC diagnosis.

### 3.4. Clinical Parameters

Table 4 reveals the relation between clinicopathological features and circRNA. High expression of tumor-promoter circRNA was contributed to poor clinicopathological features (tumor size: OR 1.680, 95% CI 1.031, 2.738; T staging: OR 1.729, 95% CI 1.074, 2.785; metastasis of lymph nodes: OR 4.657, 95% CI 1.951, 11.112). Furthermore, low expression of tumor-suppressor circRNA implied worse TNM staging (OR 2.891, 95% CI 1.052, 7.949). Of important, there was no significant difference between the expression of circRNA and other clinicopathologic parameters, including age, gender, differentiation, and distant metastasis.

### 3.5. Overall Survival (OS)

**Figure 6:**
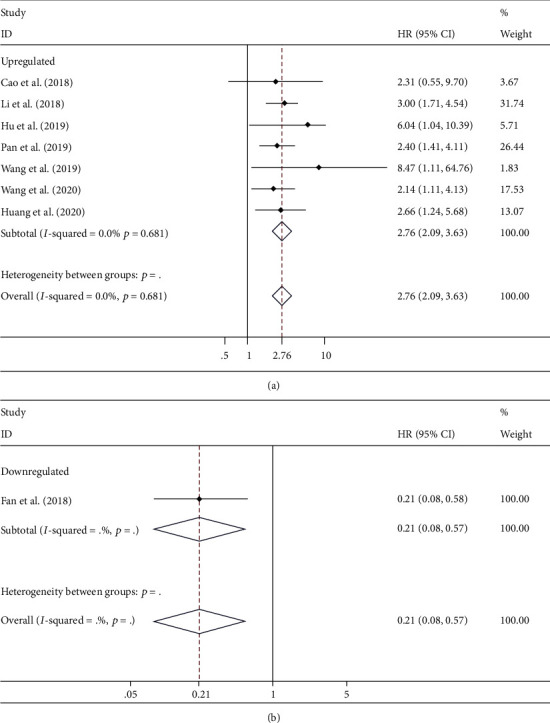
Forest plots to demonstrate that the aberrant expression of circRNAs was correlated with poor overall survival (OS) prognosis: (a) tumor-promoter circRNAs; (b) tumor-suppressor circRNAs. circRNAs, circular RNAs.

**Figure 7:**
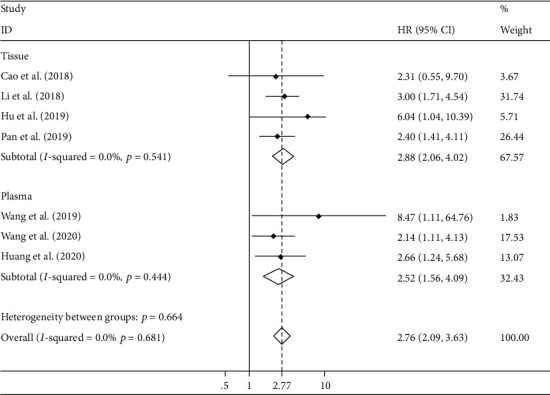
Subgroup analysis for verifying the relationship between tumor-promoter circRNA expression and overall survival prognosis. circRNAs, circular RNAs.

With no significant heterogeneity (*I*^2^ = 0%), fixed-effect models were applied to estimate the role of circRNA in OS prognosis. Upregulated tumor-promoter circRNA was correlated with worse OS (HR 2.76, 95% CI 2.09-3.63, Figure 6(a)) for EC patients. Oppositely, upregulation of tumor-suppressor circRNA notably carried out more favorable OS probability (HR 0.21, 95% CI 0.08-0.57, Figure 6(b)). Furthermore, for tumor-promoter circRNA, subgroup analysis declared that the high expression both in plasma (HR 2.52, 95% CI 1.56-4.09) and tissue (HR 2.88, 95% CI 2.06-4.02) carried out worse prognosis (Figure 7).

### 3.6. Publication Bias and Sensitivity Analysis

**Figure 8:**
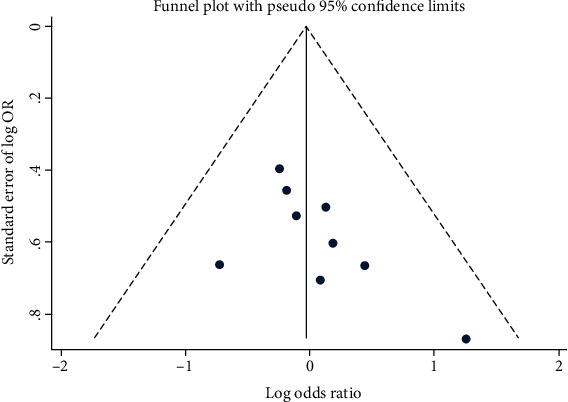
Funnel plot of circRNAs for esophageal cancer.

**Figure 9:**
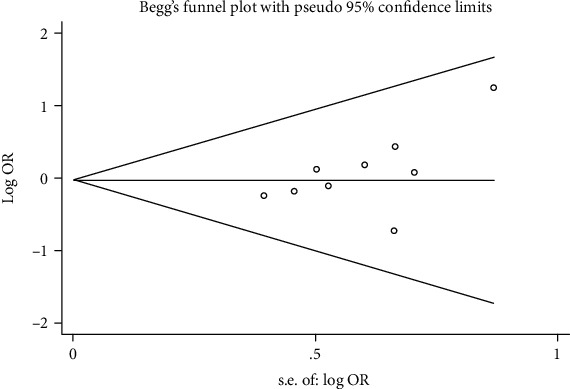
Begg's funnel plot of circRNAs for esophageal cancer.

**Figure 10:**
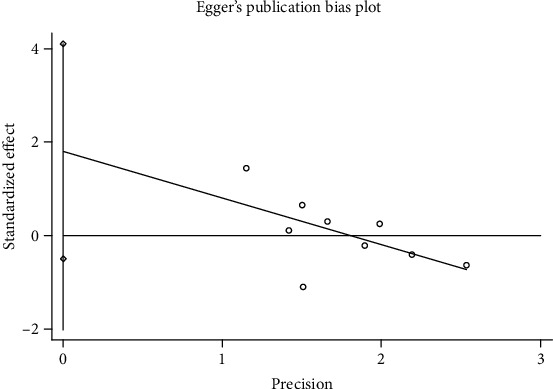
Egger's funnel plot of circRNAs for esophageal cancer.

**Figure 11:**
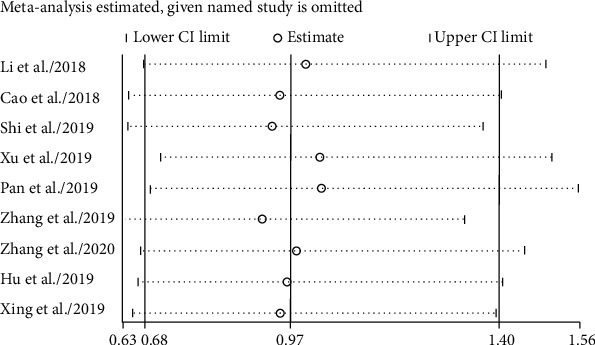
Sensitivity analysis of circRNAs for esophageal cancer.

**Figure 12:**
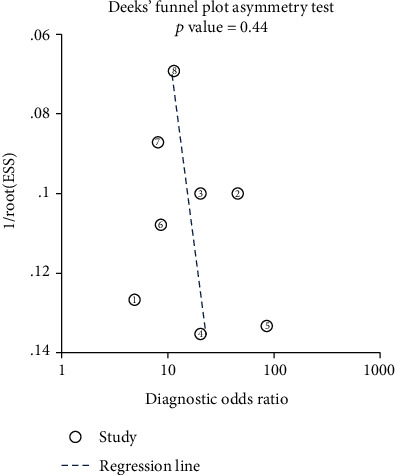
Deeks' funnel plot asymmetry test of circRNAs for esophageal cancer.

The funnel plot presented in Figure 8 demonstrated that there was no publication bias in our meta-analysis. We also performed further qualitative analysis by using Begg's test and Egger's test to evaluate the publication bias, and the results supported the conclusion that there was no publication bias (Begg's test: *p* = 0.076; Egger's test: *p* = 0.107; Figures 9 and 10). Additionally, sensitivity analysis showed that the outcomes of meta-analysis were invariable when removed the studies one by one, which concluded that the pooled outcomes were stable (Figure 11). What is more, no evidence of publication bias was implied by developing Deeks' funnel plot asymmetry test (*p* = 0.44; Figure 12).

## 4. Discussion

circRNA might be a novel tumor biomarker. Its predictive value in diagnosis and prognosis for malignancy has been gradually explored. Several circRNAs have been certified to be associated with the development and progression of various tumors, such as ciRs-7 [34]. The predictive role of circRNA in different malignancies, including lung cancer, colorectal cancer, and laryngeal cancer, has also been reported recently [35–37]. Niu et al. [38] conducted a meta-analysis to investigate the diagnostic role of circRNA in EC, which illustrated that circRNA had a favorable biological value for EC diagnosis. However, the number of studies included was small, and the relation between circRNA expression and prognosis or clinicopathological characteristics was not investigated. To our knowledge, this is the first meta-analysis involving the relationship between circRNA expression and diagnosis, prognosis, and clinicopathological characteristics of EC. In our analysis, sensitivity and specificity of circRNA for diagnosis were 0.77 and 0.80, respectively, and the AUC was 0.85. In addition, the overall DOR was 13.71, while incorporated PLR and NLR were 3.92 and 0.29, respectively. In other words, patients with the aberrant expression of circRNA were 3.92 times more likely to develop EC compared with the general population, and the proportion of patients with the normal circRNA expression only accounted for 29%. Upregulation of oncogenic circRNA was obviously associated with lymph node metastasis, tumor size, and T classification. Upregulation of downregulation of tumor-suppressor circRNA contributed to poor TNM stage. As for prognostic value, the abnormal expression of circRNA was closely associated with poor OS. Of course, just as the downregulation of miR-20b, miR-27a, and miR-181a leads to the upregulation of drug-resistant genes in gastric cancer to affect the sensitive of chemotherapy, we also expect circRNA to serve for the precise and individualized treatment of EC [39]. This is an attractive challenge that requires more clinical trials.

We investigated the diagnostic value of circRNA for EC. Due to the anomalous expression of circRNA in plasma, it is easy to obtain samples for testing when a person was suspected of suffering from EC. Meanwhile, stable structures and conservative sequences guarantee that circRNA is not prone to denature. The expression of circRNA from preoperative plasma or postoperative tumor tissue is a powerful supplement to the assessment of patient's prognosis. Our meta-analysis comprised 15 studies involving 1032 patients, which strongly manifested the function of circRNA in diagnosis, prognosis, and clinicopathological relevance for EC. It is expected that more investigations will be performed to further confirm our results, especially on tumor-suppressor circRNA.

Our analysis was developed based on PRISMA guidelines strictly and was accomplished by independent researchers utilizing appropriate retrieval strategies. We screened the studies in compliance with the rigorous inclusion and exclusion criteria. For statistical analyses, we applied precise and appropriate statistical methods, and the statistical outcomes were analyzed and interpreted carefully. Nevertheless, there were still some limitations in our study. First, the number of studies included was relatively small, especially the studies on tumor-suppressor circRNA. In order to further ascertain the results, more studies are necessary to perform in the future. Second, all studies were from China, indicating that studies on other races were needed. In addition, some studies did not provide clear sensitivity, specificity, or HR. We extracted indispensable data from supplied ROC curves and KM curves, which may lead to potential bias. Finally, we analyzed the prognostic role of circRNA by using HR, which was provided in each research via univariate analysis. The tests performed may be statistically significant but biologically less relevant if placed into a more complex context. As a result, an HR obtained from multivariate will be more credible. The prognostic role of circRNA after adjusting for other prognostic factors remains to be further explored.

## 5. Conclusion

In summary, our meta-analysis declared that the expression of circRNA in plasma had a certain value in the differential diagnosis of EC. Meanwhile, the aberrant expression of circRNA both in malignancy tissue and plasma indicated worse prognosis. circRNA might be a promising biomarker, and further researches are needed to verify its role in EC.

## Figures and Tables

**Table 1:** Main characteristics of studies for diagnosis analysis.

Study	Year	circRNA	Cancer type	Sample num		Method	Regulation	Diagnosis power		
				Case	Control			Sen.	Spe.	AUC.
Rong et al. [22]	2018	circ-DLG1	EC	35	28	qRT-PCR	Upregulated	82.86%	50.00%	0.648
Fan et al. [19]	2018	circ_0062459	EC	50	50	qRT-PCR	Downregulated	64.00%	92.00%	0.836
	2018	circ_0001946	EC	50	50	qRT-PCR	Downregulated	92.00%	80.00%	0.894
Wang (1) et al. [24]	2019	circ-TTC17	EC	30	25	qRT-PCR	Upregulated	73.33%	88.00%	0.82
Zhang et al. [25]	2019	circ-SMAD7	EC	32	25	qRT-PCR	Upregulated	78.13%	96.00%	0.859
Hu et al. [20]	2019	circ-GSK3*β*	EC	43	53	qRT-PCR	Upregulated	68.75%	81.25%	0.793
Wang (2) et al. [23]	2020	circ-SLC7A5	EC	87	53	qRT-PCR	Upregulated	67.82%	79.25%	0.772
Huang et al. [21]	2020	circ_0004771	EC	105	105	qRT-PCR	Upregulated	71.43%	81.90%	0.816

AUC, area under ROC curve; qRT-PCR, quantitative real-time polymerase chain reaction; Sen, sensitivity; Spe., specificity; EC, esophageal cancer; circRNA, circular RNA.

**Table 2:** Main characteristics of studies for prognosis analysis.

Study	Year	circRNA	Cancer type	circRNA expression		Species	Detection method	Regulation	Follow-up (months)
				High	Low				
Fan et al. [19]	2018	circ_0001946	EC	25	25	Tissue	qRT-PCR	Downregulated	33
Cao et al. [26]	2018	circ_100876	EC	37	37	Tissue	qRT-PCR	Upregulated	55
Li et al. [27]	2018	circ-CIRS7	EC	61	62	Tissue	qRT-PCR	Upregulated	90
Hu et al. [20]	2019	circ-GSK3*β*	EC	35	15	Tissue	qRT-PCR	Upregulated	21
Pan et al. [28]	2018	circ_0006948	EC	77	76	Tissue	qRT-PCR	Upregulated	60
Wang et al. [24]	2019	circ-TTC17	EC	22	8	Plasma	qRT-PCR	Upregulated	20
Wang et al. [23]	2020	circ-SLC7A5	EC	44	43	Plasma	qRT-PCR	Upregulated	40
Huang et al. [21]	2020	circ_0004771	EC	53	52	Plasma	qRT-PCR	Upregulated	48

EC, esophageal cancer; qRT-PCR, quantitative real-time polymerase chain reaction; circRNA, circular RNA.

**Table 3:** Quality assessment of eligible studies (Newcastle-Ottawa Scale).

Study	Selection			Comparability			Outcome		Total
	Adequacy of case definition	Number of case	Representativeness of the cases	Ascertainment of relevant cancers	Ascertainment of detection method	circRNA expression	Assessment of outcome	Adequate follow-up	
Huang et al.	1	1	1	1	1	1	1	1	8
Cao et al.	1	1	1	1	1	1	1	1	8
Fan et al.	1	1	1	1	1	1	1	1	8
Hu et al.	1	1	1	1	1	1	1	1	8
Li et al.	1	1	1	1	1	1	1	1	8
Pan et al.	1	1	1	1	1	1	1	1	7
Rong et al.	1	1	1	1	1	1	1	0	7
Shi et al.	1	1	1	1	1	1	0	0	6
Wang et al.	1	1	1	1	1	1	1	1	8
Wang et al.	1	1	1	1	1	1	1	1	8
Xing et al.	1	1	1	1	1	1	0	0	6
Xu et al.	1	1	1	1	1	1	0	0	6
Zhang et al.	1	1	1	1	1	1	0	0	6
Zhang et al.	1	1	1	1	1	1	1	0	7
Zhang et al.	1	1	1	1	1	1	0	0	6

**Table 4:** Clinical parameters of circRNAs in esophageal cancer.

Parameters	Tumor promoter			Tumor suppressor		
	OR	95% CI	*p*	OR	95% CI	*p*
Age (old/young)	1.096	(0.701, 1.713)	0.688	1.147	(0.427, 3.083)	0.786
Gender (M/W)	0.961	(0.660, 1.399)	0.835	1.201	(0.369, 3.909)	0.761
Tumor size (large/small)	**1.68**	**(1.031, 2.738)**	**0.037**	2.214	(0.792, 6.190)	0.13
Differentiation grade	1.02	(0.673, 1.545)	0.925	2.708	(0.559, 13.115)	0.216
TNM stage (III + IV/I + II)	2.214	(0.713, 6.876)	0.169	**2.891**	**(1.052, 7.949)**	**0.04**
T classification (T3 + T4/T1 + T2)	**1.729**	**(1.074, 2.785)**	**0.024**	—	—	—
Lymph node metastasis (Y/N)	**4.657**	**(1.951, 11.112)**	**0.001**	—	—	—
Distant metastasis (Y/N)	8.47	(0.594, 120.694)	0.115	—	—	—

CI, confidence interval; M, men; N, no; W, women; Y, yes; OR, odds ratio; na, not available. The results are in bold if *p* < 0.05.

## Data Availability

The data used to support the findings of this study are included within the article.

## Supplementary Materials

Supplementary Materials The authors have completed the PRIMSA reporting checklist.
