# Supplementary material for: Prognostic and Diagnostic Significance of circRNA Expression in Esophageal Cancer: A Meta-analysis
Source: Gastroenterol Res Pract. 2020 Dec 1;2020:8437250. doi: 10.1155/2020/8437250 (PMC7723476; doi:10.1155/2020/8437250)
Supplement: Supplementary Materials — The authors have completed the PRIMSA reporting checklist. [file 8437250.f1.doc]

| **Section/topic** | **#** | **Checklist item** | **Reported on page #** |
| --- | --- | --- | --- |
| **TITLE** | | | 1 |
| Title | 1 | Prognostic and diagnostic value of circRNAs expression in esophageal carcinoma: a meta-analysis | 1 |
| **ABSTRACT** | | | 2 |
| Structured summary | 2 | Background; Methods; Results; Conclusions; Keywords; Key points | 2 |
| **INTRODUCTION** | | | 3 |
| Rationale | 3 | Introduction | 3 |
| Objectives | 4 | Introduction | 3 |
| **METHODS** | | | 4-5 |
| Protocol and registration | 5 |  |  |
| Eligibility criteria | 6 | Search Strategy | 4 |
| Information sources | 7 | Search Strategy | 4 |
| Search | 8 | Search Strategy | 4 |
| Study selection | 9 | Study selection, Data Extraction and Quality Assessment | 4 |
| Data collection process | 10 | Data Extraction and Quality Assessment | 4-5 |
| Data items | 11 | Statistical analysis | 5 |
| Risk of bias in individual studies | 12 | Statistical analysis | 5 |
| Summary measures | 13 | Statistical analysis | 5 |
| Synthesis of results | 14 | Statistical analysis | 5 |

Page 1 of 2

| **Section/topic** | **#** | **Checklist item** | **Reported on page #** |
| --- | --- | --- | --- |
| Risk of bias across studies | 15 | Statistical analysis | 5 |
| Additional analyses | 16 |  |  |
| **RESULTS** | | | 6-8 |
| Study selection | 17 | Search Results | 6 |
| Study characteristics | 18 | Study characteristics | 6 |
| Risk of bias within studies | 19 | Study characteristics | 6 |
| Results of individual studies | 20 | Study characteristics | 6 |
| Synthesis of results | 21 | Diagnosis analysis; Clinicopathological parameters; Overall survival | 6-7 |
| Risk of bias across studies | 22 | Publication bias and sensitivity analysis | 8 |
| Additional analysis | 23 |  |  |
| **DISCUSSION** | | | 8-10 |
| Summary of evidence | 24 | Discussion | 8-9 |
| Limitations | 25 | Discussion | 9-10 |
| Conclusions | 26 | Conclusions | 10 |
| **FUNDING** | | | 10 |
| Funding | 27 |  | 10 |

*From:*  Moher D, Liberati A, Tetzlaff J, Altman DG, The PRISMA Group (2009). Preferred Reporting Items for Systematic Reviews and Meta-Analyses: The PRISMA Statement. PLoS Med 6(7): e1000097. doi:10.1371/journal.pmed1000097

For more information, visit: **www.prisma-statement.org**.

Page 2 of 2
